# The impact of temporal framing of breast cancer risk on perceptions of and motivations to engage with information about early diagnosis: Evidence from an online experiment

**DOI:** 10.1371/journal.pone.0320245

**Published:** 2025-03-26

**Authors:** Sandro Tiziano Stoffel, Camilla Natale, Christian von Wagner

**Affiliations:** 1 Research Department of Behavioural Science and Health, UCL, London, United Kingdom; 2 Institute of Pharmaceutical Medicine (ECPM), University of Basel, Basel, Switzerland; University of Hull, UNITED KINGDOM OF GREAT BRITAIN AND NORTHERN IRELAND

## Abstract

This study investigates the application of Construal Level Theory (CLT) to grasp how individuals perceive and respond to breast cancer risk in both near and distant future scenarios. Employing a two-stage methodology, we initially conducted a preliminary survey with 201 women aged 40 to 50, evaluating their perceptions of various phrasings of breast cancer risk information, including ‘1 in x’, ‘x% of’, and ‘x-y% probability’. Subsequently, an online experiment involving 1052 women in the same age group explored the influence of temporal framing on perceived risk and intentions for breast self-checks. We selected the most understandable, imaginable, and motivational phrasing from the preliminary survey for the experiment. The participants were divided into two groups: near-future framing (N = 526) and distant-future framing of developing breast cancer (N = 526). Study 1 revealed that women found the ‘1 in x’ framing to be the easiest to understand, imagine, and most motivational. However, the subsequent experiment (Study 2) did not find any significant effects of temporal framing on women’s perceived risk of developing breast cancer, perceived importance of self-checks, intention to conduct self-checks, or interest in learning more about self-checks. Nonetheless, it was noteworthy that individuals exposed to near-future framing perceived their risk as closer in time compared to those presented with distant-future framing (OR = 1.42, 95% CI: 1.15-1.77 p = 0.001; aOR = 1.42, 95% CI: 1.14-1.76; p = 0.002). In conclusion, our study found that temporal distance of breast cancer risk doesn’t affect risk perception or information-seeking behaviour, suggesting a focus on clear, motivational risk communication rather than temporal framing alone.

## Introduction

Breast cancer is a significant global concern, comprising 15% of new cancer cases [1]. It ranks as the fourth leading cause of cancer-related death in the UK, with mortality rates decreasing by 18% in the last decade due to improved early detection and diagnostics [1]. Early diagnosis substantially improves outcomes, reducing invasive procedures and preserving breast tissue [2]. Emphasizing prevention and awareness is vital, and behavioural science methods aid in effective public and high-risk individual communication. The Theory of Planned Behaviour (TPB) posits that intentions and behaviour are influenced by the perceived importance of cancer prevention, which in turn is influenced by the perceived risk [3]. Behavioural studies have shown that the way health risks are framed significantly influences individuals’ perception of their severity and likelihood [4,5]. Temporal framing, a type of risk framing, demonstrates that people perceive immediate health risks as more severe and urgent than those in the distant future [6,7]. Studies further indicate that individuals prioritize immediate concerns over future ones [8]. This can be explained by **Construal** Level Theory (CLT), hyperbolic discounting, and present bias [6,9,10]. CLT introduces the concept of psychological distance and states that distant-future events are perceived more abstractly than near-future ones [6,7], and concrete risks are perceived as riskier [11]. Hyperbolic discounting states that immediate risks (short-term) may be perceived as more urgent because they heavily discount the future [9,12]. Similarly, present bias leads individuals to prioritize immediate over long-term risks [10]. These theories suggest that the temporal framing of breast cancer risk influences women’s breast cancer risk perception and their intentions to engage in preventive behaviours. Temporal framing effects on health risk perception have been observed in heart diseases [11], healthy eating [13], virus threats [14], smoking [15], and colorectal cancer screening [16]. In Kim and Kim’s study, smokers perceived the risk of a heart attack, which is temporally closer, as more personally relevant and susceptible compared to the more distant risk of larynx cancer [15]. With higher perceived health risks, individuals’ attitudes towards preventive behaviours improve, and their intentions to engage in these actions increase [3,15]. This is because recognizing a significant health risk makes preventive measures seem more necessary and beneficial, motivating individuals to adopt behaviours that can reduce the risk.

In this study, we aimed to test whether communicating more imminent breast cancer risk increases intentions to engage in preventive health behaviours in a healthy sample of women, while keeping the overall health risk constant. Specifically, we investigated the perceived risk of developing breast cancer in the next 1-10 years versus the next 11-20 years, examining the trade-off between lower risk at an earlier stage and higher risk at a later stage. Consistent with CLT and hyperbolic discounting, we hypothesized that women would perceive the lower risk of developing breast cancer in the next 1-10 years as higher than the more distant, but higher risk of developing breast cancer in the next 11-20 years.

## Methods

To test this hypothesis, we conducted a two-stage study. In the first stage (preliminary survey), we evaluated women’s understanding and motivation concerning different risk information phrasings (i.e., “1 in x,” “x% of,” “x% probability”). In the subsequent experiment, we presented women with either near-future or distant-future frames of the most comprehensible phrasing identified earlier. We then measured their perceived breast cancer risk, intention to perform breast self-checks, and active interest in learning more about them.

### Study design

Both the preliminary and the experiment surveys were conducted online between August 4^th^ and 25^th^, 2023 using Qualtrics. The sample consisted of cisgender women, aged 40-50, living in England, who were aware of their menopausal status and had no previous diagnosis of breast cancer. The study participants were recruited through the online platform Prolific. The gender-specific selection was based on women’s higher susceptibility to breast cancer [17]. Breast cancer incidence data were sourced from the Cancer Research UK website [1], covering the years 2014 to 2016. The risk of developing breast cancer was then calculated for women aged 40-50 and 60-70. All participants received information about the study’s objectives and were offered £0.50 as compensation for completing the survey. Ineligible and disinterested individuals were excluded from the study. Prolific IDs were used to ensure each individual could only participate in one survey.

The study obtained approval from the UCL Research Ethics Committee (26035/001). All study participants were asked to provide written consent to participate in the study and to have their data included and published in scientific publications.

### Study 1 - Preliminary survey

The primary objective of the preliminary online survey was to determine the most comprehensible framing of breast cancer risk for the experiment. For this, the survey presented study participants with three breast cancer risk statements for women aged 60-70 (see Table 1) in random order and then asked them to assess them in terms of ease of understanding, imagination, motivation for preventive behaviours, and suitability for public communication (see Text S1 in the supplementary file for the questionnaire).

**Table 1:** Breast cancer risk messages presented to women in Study 1.

1 in x	1 in 29 women between the age of 60 and 70 is diagnosed with breast cancer.
x% of.	43% of women diagnosed with breast cancer were older than 65 years.
x-y% probability	There is a 2-3% probability of developing breast cancer in the next 20 years.

Specifically, study participants were asked to respond to the following questions: (1) ‘Given that all three messages refer to the same risk of women being diagnosed with breast cancer, which message is easier to understand?’, (2) ‘Given that all three messages refer to the same risk of women being diagnosed with breast cancer, which is easiest to imagine?’, (3) ‘Given that all three messages refer to the same risk of women being diagnosed with breast cancer, which is more motivational for preventing breast cancer?’, and (4) ‘Given that all three messages refer to the same risk of women being diagnosed with breast cancer, which should the NHS use for communication?’

We also included a numeracy skill question adapted from Lipkus and colleagues [18], and gathered demographic information regarding education, employment, marital status, and ethnicity. Numeracy skills were assessed with the question: ‘Which number represents the highest disease risk?’ with the options: ‘1/10,’ ‘1/100,’ ‘1/1000,’ and ‘Don’t know’ [18]. All responses to the questionnaire were analysed descriptively, using frequency tables and figures.

### Study 2 – Experiment

The primary objective of Study 2 was to assess the impact of near-future and distant-future breast cancer risk messages on participants’ perceived risk of developing cancer and their intentions to perform breast self-checks. The experiment began with participant information and the same three filter questions and one numeracy question as in the preliminary survey (see Text S2 in the supplementary file for the questionnaire). They were then asked three questions about their subjective and objective knowledge of breast cancer risk factors, as well as their information-seeking behaviour regarding breast cancer prevention. Following this, the survey software randomly assigned each study participant to one of two experimental conditions. Individuals in the near-future condition received the information about the risk of being diagnosed with breast cancer between ages 40 and 50, while those in the distant-future condition received the information about the risk of being diagnosed with breast cancer between ages 60 and 70. Participants were then asked about their perceived temporal proximity of breast cancer risk using the question: ‘How imminent or distant do you believe the likelihood of developing breast cancer is for you?’ This question served as a manipulation check. They also assessed the seriousness of breast cancer health consequences with the question: ‘How serious do you consider the health consequences of breast cancer to be?’ Both questions used a partially labelled 7-point Likert scale response (1 =  very imminent vs. 7 =  very distant for the first question and 1 =  not serious at all vs. 7 =  very serious for the second), adapted from prior research [15].

Next, study participants were asked about their perceived importance of breast self-exams using the question: ‘A breast self-exam can help you detect breast cancer earlier when it is easier to treat. In the context of this information, how important is it for you to learn how to conduct a breast self-exam?’ Participants selected from five responses, ranging from ‘Not important at all’ to ‘Extremely important.’ Intentions for breast self-checks were assessed with the question: ‘How often would you be willing to a conduct breast self-examination?’ Choices included ‘never,’ ‘once a year,’ ‘once every 6 months,’ ‘once a month,’ and ‘every day.’

Active interest was operationalized as the decision to read further information about breast self-checks, rather than skipping that section [19]. The question featured response options adapted from previous studies [16,20], offering participants the choice to ‘read information on the next page before continuing with the survey’ or ‘skip information on the next page and continue with the survey.’ Those choosing to read were subsequently asked three multiple-choice comprehension questions to gauge their engagement with the information. Similar to the preliminary survey, the experiment concluded with demographic questions about participants’ education, employment, living status, and ethnicity. The only difference was the addition of two questions about family history of breast cancer. Upon completing the questionnaire, participants were provided with supplementary resources for further exploration.

The analysis of the experiment included chi-square tests of independence and multivariable logistic regressions. Adjusted regression models incorporated covariates such as age, menopause status, ethnicity, education, employment status, numeracy skills, and family history. Odds ratios (ORs) and 95% confidence intervals (CIs) for the experimental conditions are reported in the text, while comprehensive models displaying all covariates are available in the supplementary file. To ensure adequate statistical power, we determined the sample size for our experiment in advance. We aimed for an 80% chance of detecting a medium effect size (d =  0.5), which translates to a difference of approximately 0.5 points on the 7-point Likert scale used to measure risk perception, with a significance level (alpha) of 0.05, based on prior research [15,21]. Using a t-test for independent samples, we estimated that we would need at least 520 participants in each experimental condition.

## Results

### Study 1 - Preliminary survey

Out of the initial 238 consenting participants, 34 were ineligible, and 3 dropped out during the survey. This resulted in a final sample of 201 participants, predominantly aged between 40 and 45 (57.2%), identifying as white British (83.6%), and being pre-menopausal (88.6%). A considerable portion were in paid employment (83.1%) and in a partnership or married (75.6%). Most had completed A-levels (82.6%) and answered the numeracy question correctly (85.1%, see Table S1 in the supplementary file).

**Fig 1:**
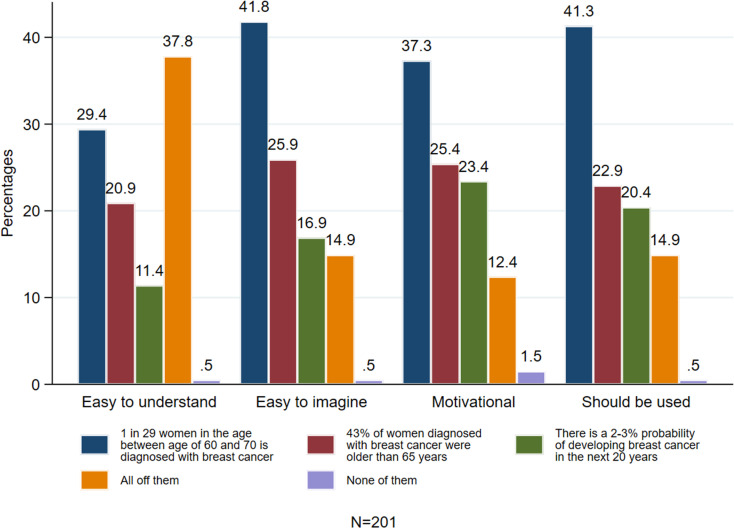
Perception of the messages in the preliminary survey.

Examining which breast cancer risk message was perceived as easiest to understand and imagine, Fig 1 reveals that most participants (37.8%) found all messages easy to understand. Among participants, the ‘1 in x’ format was deemed the easiest to understand by 29.4% and the easiest to imagine by 41.8%. Additionally, most participants (37.3%) found this phrasing motivating for engaging with breast cancer prevention information, and 41.3% of participants recommended the National Health Service (NHS) use this phrasing for information delivery, highlighting its perceived effectiveness. Consequently, the “1 in x” phrasing was selected for the experiment to investigate the impact of temporal distance on women’s breast cancer risk perception and their intentions regarding self-checks and seeking further information. Specifically, the message in the near-future condition stated that ‘1 in 69 women are diagnosed with breast cancer between ages 40 and 50,’ while the message in the distant-future condition read that ‘1 in 29 women is diagnosed with breast cancer between ages 60 and 70. ‘

### Study 2 - Experiment

**Fig 2:**
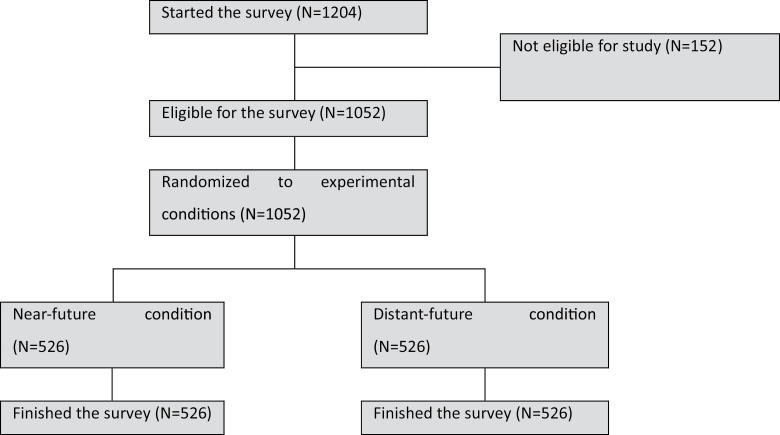
Flow chart though study 2.

Fig 2 shows that out of the initial 1,204 study participants who initiated the survey, 152 were deemed ineligible, resulting in a study sample of 1,052 women, with 526 in each condition. Sociodemographic characteristics resembled those in Study 1, with the majority falling within the 40-45 age group (56.9%), being pre-menopausal (91.4%), identifying as white British (84.5%), holding A-levels or higher (87.2%), answering the numeracy question correctly (82.9%), and lacking a family history of breast cancer (69.8%). Sociodemographic characteristics across the two experimental conditions were balanced, except for numeracy skills (see Table S2 in the supplementary file).

**Fig 3:**
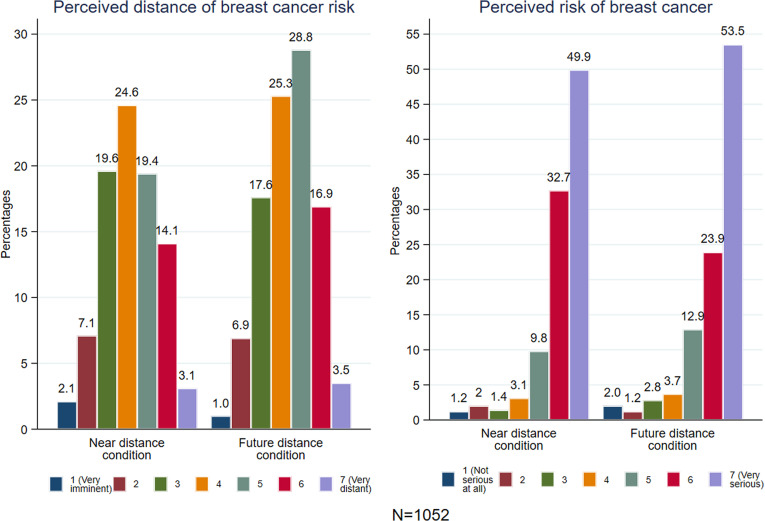
Effect of the messages on perceived temporal distance and risk of breast cancer in study 2.

Consistent with the working hypothesis, Fig 3 illustrates that women in the near-future condition perceived breast cancer risk as significantly more imminent compared to those in the distant-future condition (χ2(6, N =  1052) =  22.91, p < 0.001). This observation aligns with the results of the ordinal logistic regression for perceived distance presented in Table S3 in the supplementary file (OR 1.42, 95%CI 1.15-1.77, p = 0.001; aOR 1.42, 95%CI 1.14-1.76, p = 0.002). While temporal distance was associated with perceived seriousness (χ2(6, N =  1052) =  21.68, p < 0.001), the regressions suggest a non-significant opposite effect (OR 0.81, 95%CI 0.65-1.02, p = 0.071; aOR 0.82, 95%CI 0.66-1.04, p = 0.108).

Furthermore, Table S4 and Fig S1 in the supplementary file show that the temporal framing was not associated with perceived importance of self-checks (OR 0.90, 95%CI 0.70-1.16, p = 0.416; aOR 0.91, 95%CI 0.70-1.17, p = 0.459), intentions for self-checks (OR 0.89, 95% CI 0.69-1.16, p =  0.384; aOR 0.87, 95% CI 0.67-1.14, p =  0.321), or interest in learning more (OR 1.06, 95% CI 0.83-1.35, p =  0.659; aOR =  1.07, 95% CI 0.83-1.38, p =  0.586). Most women (70.6%) intended to perform monthly breast self-checks, with 60.5% wanting additional information (59.9% in the near-future condition vs. 61.2% in the distant-future condition, χ²(6, N =  1052) =  0.19, p =  0.659). Among them, 85.5% correctly answered all three comprehension questions, while a few got one (13.8%) or two (0.8%) questions wrong, and none answered all incorrectly. Responses did not significantly differ between the two conditions (Fisher’s exact test, p =  0.215).

## Discussion

This study tested the temporal distance of breast cancer risk on women’s perceived risk and intentions to read more about and perform breast self-checks. CLT explains how different types of psychological distance—such as time, space, social relationships, or hypothetical scenarios—affect how individuals perceive health risks [6,7]. Health risks that are far in the future should be perceived as more abstract and, therefore, less severe than those that are closer in time. However, contrary to what CLT predicts, our study did not find that a closer temporal framing of the risk of developing breast cancer amplifies the perceived severity or intentions to engage in preventive behaviours [13,15,22,23]. One potential reason we were unable to replicate prior findings is that, in order to manipulate temporal framing, we had to adjust the probability of the health risk occurring. We did this to ensure that study participants received accurate probabilities of developing breast cancer. Although the risk of developing breast cancer between ages 60 and 70 is more distant than the risk between ages 40 and 50, the former carries a higher probability due to the age-related increase in cancer risk. This trade-off could explain why study participants perceived the nearer risk as closer in time but not more serious. According to hyperbolic discounting, there exists a switching point—a specific probability threshold—beyond which individuals perceive the near-future risk as higher than the distant-future risk. This means that even if the distant-future risk has a higher overall probability (due to age-related factors), people may still perceive the near-future risk as more urgent and concerning. The results of our experiment suggest, although not statistically significant, that study participants perceived the future risk as slightly higher. This implies that the immediate risk was too low for the given temporal distance. Alternatively, it could have been caused by the ratio bias, which is individuals’ tendency to judge a probability by the numerator instead of the overall proportion [24,25]. Thus, study participants may have focused more on the numerators (1 in 29 and 1 in 69) and less on the denominators (11-20 years and 1-10 years).

A further explanation could be that study participants had to trade-off risk and temporal distance, which required numeracy skills. Study participants in our sample demonstrated a high level of numeracy. Unlike a prior study that observed an effect of temporal framing [16], the majority of our participants correctly answered the numeracy question. Consequently, the women in our study may not have encountered difficulties with numerical risk assessments [26,27].

Our study offers several advantages over previous research on temporal framing. First, we only changed the timing of the health risk, but not the risk itself. This means that study participants were told breast cancer could develop either sooner or later. Second, the experiment featured a large analytical sample with statistical power. Third, our perception and intention questions were adapted from previous studies, and we included a behavioural outcome measure in the form of active interest in reading more about breast self-checks, aiming to bridge the intention-behaviour gap. Although the vast majority of women stated that they would perform breast self-checks every month, the literature on the intention-behaviour gap suggests that these intentions do not necessarily translate into behaviour [28]. Finally, even though we did not observe an effect of temporal framing on perceived risk, the results suggest that women perceived the risk as temporally closer in the near-distant condition, indicating that the manipulation was successful.

This study has some important limitations, which call for follow-up research. First, as previously mentioned, in order to manipulate the temporal framing of developing breast cancer, we had to adapt its probability of occurrence, introducing a further complication. Furthermore, the experiment measured only risk perception and intentions to engage in preventive behaviour for the specific time frame and risk. In future studies, an alternative study design featuring the dynamic staircase method could identify the time frame and risk at which individuals perceive the more distant health risk as higher [29]. This study design would involve presenting individuals with a series of binary comparisons between nearer and more distant risks of developing breast cancer [30]. Participants would make choices between an imminent and a distant health risk based on which they perceive as more serious. If the participant perceives the initial imminent risk as more serious, the next task would present a slightly lower imminent risk. This process continues until the study participant perceives the distant risk as more serious, allowing researchers to determine the level at which the imminent risk is considered less severe than the distant risk. Secondly, although we inquired with study participants about their family history and how frequently they consider the implications of their health-related behaviours and their family’s cancer history, we did not evaluate their susceptibility to the risk of breast cancer, which is a crucial aspect [31]. Therefore, future studies should also examine individual factors, including personality traits. Similarly, we did not include the validated Breast Cancer Awareness Measure before the experimental manipulation to assess participants’ current knowledge and behaviour regarding breast self-checks. Its inclusion could have provided insights into changes in risk perception [32]. Third, our risk messages and questionnaire were not co-developed with public and patient representatives. As a result, the information may not have been communicated accurately, or the questions may have been too difficult for the participants. Therefore, we recommend involving patients in the development of risk communication in future studies. Fourth, our study sample was not representative because it mainly included participants with White ethnic background and who had at least A levels in education. This indicates that they likely had strong English language skills, but we did not control for their first language. Non-native English speakers might have had difficulty understanding the risk information. Additionally, since study participants self-selected to participate, this could have introduced selection bias. Future research should include a language assessment and explore these findings in a more representative population. Fifth, our study focused only on one specific health risk, breast cancer in our case, so the findings may not apply to other health risks. Future studies could investigate temporal framing for various health issues. In terms of outcome measures, self-examinations are just one aspect of breast cancer prevention, and considering other preventive behaviours like weight management, smoking cessation, or alcohol abstinence could have enhanced the findings. Furthermore, our intention questions might have been influenced by social desirability. Similarly, a different behavioural outcome measure may have been better suited. Finally, our investigation of temporal framing focused on numerical risk; future research could test alternative presentations, such as visuals, graphics, narratives or frequency labels.

## Conclusion

In conclusion, this study found that while temporal framing influenced perceptions of the temporal proximity of breast cancer, it did not significantly affect motivations to engage with preventive information or the seriousness attributed to breast cancer. This suggests that a focus on clear, motivational risk communication is more effective than relying on temporal framing alone.

## Supporting information

Table S1 Description of the study sample in Study 1 (N = 201). (DOCX)

Table S2 Description of the study sample’s demographics in Study 2 (N = 1052). (DOCX)

Table S3 Ordinal logistic regression on perception items (N = 1052). (DOCX)

Table S4 Logistic regressions on intentions and active interest (N = 1052). (DOCX)

Text S1 Preliminary questionnaire. (DOCX)

Text S2 Main questionnaire. (DOCX)
