# Supplementary material for: The impact of temporal framing of breast cancer risk on perceptions of and motivations to engage with information about early diagnosis: Evidence from an online experiment
Source: PLoS One. 2025 Mar 26;20(3):e0320245. doi: 10.1371/journal.pone.0320245 (PMC11940651; doi:10.1371/journal.pone.0320245)
Supplement: Table S1 — (DOCX) [file pone.0320245.s001.docx]

Table S1. Description of the study sample in Study 1 (N=201)

|  | | N | (%) |
| --- | --- | --- | --- |
| Age | |  |  |
|  | 40-45 years old | 115 | (57.2) |
|  | 46-50 years old | 86 | (42.8) |
| Menopausal status | |  |  |
|  | Premenopausal | 178 | (88.6) |
|  | Postmenopausal | 23 | (11.4) |
| Numeracy question | |  |  |
|  | Wrong | 30 | (14.9) |
|  | Right | 171 | (85.1) |
| Education level | |  |  |
|  | No A-levels | 35 | (17.4) |
|  | A-levels or above | 166 | (82.6) |
| Paid employment | |  |  |
|  | No | 34 | (16.9) |
|  | Yes | 167 | (83.1) |
| Marital status | |  |  |
|  | Single, divorced, separated or widowed | 49 | (24.4) |
|  | Married or living with partner | 152 | (75.6) |
| Ethnicity | |  |  |
|  | White British | 168 | (83.6) |
|  | Other White background | 11 | (5.4) |
|  | Asian background | 10 | (5.0) |
|  | African/Black background | 5 | (2.5) |
|  | Mixed or other background | 7 | (3.5) |
