# Supplementary material for: The impact of temporal framing of breast cancer risk on perceptions of and motivations to engage with information about early diagnosis: Evidence from an online experiment
Source: PLoS One. 2025 Mar 26;20(3):e0320245. doi: 10.1371/journal.pone.0320245 (PMC11940651; doi:10.1371/journal.pone.0320245)
Supplement: Table S2 — (DOCX) [file pone.0320245.s002.docx]

Table S2. Description of the study sample’s demographics in Study 2 (N=1052)

|  |  | Near future  (N=526) | | Distant future  (N=526) | | p-value* | Total  (N=1052) | |
| --- | --- | --- | --- | --- | --- | --- | --- | --- |
|  | | N | (%) | N | (%) |  | N | (%) |
| Age | |  |  |  |  |  |  |  |
|  | 40-45 years old | 288 | (54.7) | 311 | (59.1) | 0.152 | 599 | (56.9) |
|  | 46-50 years old | 238 | (45.3) | 215 | (40.9) |  | 453 | (43.1) |
| Menopausal status | |  |  |  |  |  |  |  |
|  | Premenopausal | 475 | (90.3) | 486 | (92.4) | 0.228 | 961 | (91.4) |
|  | Postmenopausal | 51 | (9.7) | 40 | (7.6) |  | 91 | (8.6) |
| Numeracy question | |  |  |  |  |  |  |  |
|  | Wrong | 76 | (14.5) | 104 | (19.8) | 0.022 | 180 | (17.1) |
|  | Right | 450 | (85.5) | 422 | (80.2) |  | 872 | (82.9) |
| Education level | |  |  |  |  |  |  |  |
|  | No A-levels | 69 | (13.1) | 66 | (12.6) | 0.782 | 135 | (12.8) |
|  | A-levels or above | 457 | (86.9) | 460 | (87.4) |  | 917 | (87.2) |
| Paid employment | |  |  |  |  |  |  |  |
|  | No | 75 | (14.3) | 80 | (15.2) | 0.664 | 155 | (14.7) |
|  | Yes | 451 | (85.7) | 446 | (84.8) |  | 897 | (85.3) |
| Marital status | |  |  |  |  |  |  |  |
|  | Single, divorced, separated or widowed | 161 | (30.6) | 167 | (31.8) | 0.690 | 328 | (31.2) |
|  | Married or living with partner | 365 | (69.4) | 359 | (68.2) |  | 724 | (68.8) |
| Ethnicity | |  |  |  |  |  |  |  |
|  | White British | 441 | (83.8) | 448 | (85.2) | 0.942 | 889 | (84.5) |
|  | Other White background | 36 | (6.8) | 30 | (5.7) |  | 66 | (6.3) |
|  | Asian background | 19 | (3.6) | 19 | (3.6) |  | 38 | (3.6) |
|  | African/Black background | 15 | (2.9) | 13 | (2.5) |  | 28 | (2.7) |
|  | Mixed or other background | 15 | (2.9) | 16 | (3.0) |  | 31 | (2.9) |
| History with breast cancer | |  |  |  |  |  |  |  |
|  | None | 375 | (71.3) | 359 | (68.2) | 0.510 | 734 | (69.8) |
|  | 1^st^ degree relative | 42 | (8.0) | 41 | (7.8) |  | 83 | (7.9) |
|  | 2^nd^ degree relative | 89 | (16.9) | 94 | (17.9) |  | 183 | (17.4) |
|  | Other | 4 | (0.8) | 7 | (1.3) |  | 11 | (1.0) |
|  | I don’t know | 16 | (3.0) | 25 | (4.8) |  | 41 | (3.9) |

* Chi Square test

* *p*<0.05; ** *p*<0.01
