# Supplementary material for: The impact of temporal framing of breast cancer risk on perceptions of and motivations to engage with information about early diagnosis: Evidence from an online experiment
Source: PLoS One. 2025 Mar 26;20(3):e0320245. doi: 10.1371/journal.pone.0320245 (PMC11940651; doi:10.1371/journal.pone.0320245)
Supplement: Table S3 — (DOCX) [file pone.0320245.s003.docx]

Table S3. Ordinal logistic regression on perception items (N=1052)

|  | Perceived temporal distance of breast cancer [1;7] | | | | |  | Perceived seriousness of breast cancer [1;7] | | | | |  | Perceived importance of self-checks [1;7] | | | | |
| --- | --- | --- | --- | --- | --- | --- | --- | --- | --- | --- | --- | --- | --- | --- | --- | --- | --- |
|  | Unadjusted model | |  | Adjusted model | |  | Unadjusted model | |  | Adjusted model | |  | Unadjusted model | |  | Adjusted model | |
|  | OR | 95% CI |  | aOR | 95% CI |  | OR | 95% CI |  | aOR | 95% CI |  | OR | 95% CI |  | aOR | 95% CI |
| Condition |  |  |  |  |  |  |  |  |  |  |  |  |  |  |  |  |  |
| Near future | Ref. |  |  | Ref |  |  | Ref. |  |  | Ref. |  |  | Ref, |  |  | Ref. |  |
| Distant future | 1.424 | 1.147 - 1.768** |  | 1.415 | 1.137 - 1.761** |  | 0.811 | 0.646 - 1.018 |  | 0.828 | 0.658 - 1.042 |  | 0.899 | 0.697 - 1.161 |  | 0.907 | 0.700 - 1.175 |
| Age |  |  |  |  |  |  |  |  |  |  |  |  |  |  |  |  |  |
| 40-45 years old | Ref. |  |  | Ref |  |  | Ref. |  |  | Ref. |  |  | Ref, |  |  | Ref. |  |
| 46-50 years old | 0.722 | 0.581 - 0.897** |  | 0.709 | 0.564 - 0.890** |  | 1.368 | 1.087 - 1.721** |  | 1.325 | 1.043 - 1.684* |  | 0.988 | 0.763 - 1.278 |  | 0.933 | 0.713 - 1.221 |
| Menopausal status |  |  |  |  |  |  |  |  |  |  |  |  |  |  |  |  |  |
| Premenopausal | Ref. |  |  | Ref |  |  | Ref. |  |  | Ref. |  |  | Ref, |  |  | Ref. |  |
| Postmenopausal | 0.821 | 0.549 - 1.226 |  | 0.957 | 0.630 - 1.453 |  | 1.507 | 0.992 - 2.288 |  | 1.288 | 0.833 - 1.991 |  | 1.507 | 0.926 - 2.454 |  | 1.502 | 0.903 - 2.497 |
| Numeracy question |  |  |  |  |  |  |  |  |  |  |  |  |  |  |  |  |  |
| Wrong | Ref. |  |  | Ref |  |  | Ref. |  |  | Ref. |  |  | Ref, |  |  | Ref. |  |
| Right | 0.797 | 0.595 - 1.067 |  | 0.819 | 0.609 - 1.102 |  | 1.070 | 0.790 - 1.449 |  | 1.177 | 0.861 - 1.609 |  | 0.828 | 0.585 - 1.171 |  | 0.910 | 0.637 - 1.299 |
| Education level |  |  |  |  |  |  |  |  |  |  |  |  |  |  |  |  |  |
| No A-levels | Ref. |  |  | Ref |  |  | Ref. |  |  | Ref. |  |  | Ref, |  |  | Ref. |  |
| A-levels or above | 1.060 | 0.775 - 1.449 |  | 1.075 | 0.779 - 1.484 |  | 0.661 | 0.460 - 0.950* |  | 0.658 | 0.452 - 0.959* |  | 0.565 | 0.368 - 0.865** |  | 0.578 | 0.373 - 0.897* |
| Paid employment |  |  |  |  |  |  |  |  |  |  |  |  |  |  |  |  |  |
| No | Ref. |  |  | Ref |  |  | Ref. |  |  | Ref. |  |  | Ref, |  |  | Ref. |  |
| Yes | 1.026 | 0.750 - 1.403 |  | 0.965 | 0.701 - 1.328 |  | 0.868 | 0.627 - 1.203 |  | 0.980 | 0.700 - 1.372 |  | 0.830 | 0.571 - 1.206 |  | 0.936 | 0.638 - 1.375 |
| Marital status |  |  |  |  |  |  |  |  |  |  |  |  |  |  |  |  |  |
| Single, divorced, separated or widowed | Ref. |  |  | Ref |  |  | Ref. |  |  | Ref. |  |  | Ref, |  |  | Ref. |  |
| Married or living with partner | 0.776 | 0.613 - 0.981* |  | 0.765 | 0.603 - 0.970* |  | 1.371 | 1.075 - 1.748* |  | 1.410 | 1.102 - 1.804** |  | 1.518 | 1.157 - 1.991** |  | 1.526 | 1.159 - 2.009** |
| Ethnicity |  |  |  |  |  |  |  |  |  |  |  |  |  |  |  |  |  |
| White British | Ref. |  |  | Ref |  |  | Ref. |  |  | Ref. |  |  | Ref, |  |  | Ref. |  |
| Other White background | 1.349 | 0.853 - 2.134 |  | 1.282 | 0.809 - 2.032 |  | 1.239 | 0.763 - 2.012 |  | 1.355 | 0.831 - 2.208 |  | 0.795 | 0.478 - 1.323 |  | 0.833 | 0.497 - 1.396 |
| Asian background | 0.770 | 0.427 - 1.390 |  | 0.682 | 0.373 - 1.249 |  | 1.110 | 0.595 - 2.071 |  | 1.086 | 0.573 - 2.060 |  | 0.976 | 0.499 - 1.911 |  | 0.957 | 0.483 - 1.897 |
| African/Black background | 1.995 | 0.948 - 4.201 |  | 1.755 | 0.826 - 3.728 |  | 2.457 | 1.076 - 5.610* |  | 2.734 | 1.182 - 6.322* |  | 2.186 | 0.823 - 5.806 |  | 2.236 | 0.832 - 6.010 |
| Mixed or other background | 0.689 | 0.363 - 1.310 |  | 0.613 | 0.319 - 1.179 |  | 1.032 | 0.522 - 2.043 |  | 1.064 | 0.533 - 2.123 |  | 1.096 | 0.517 - 2.320 |  | 1.074 | 0.502 - 2.298 |
| History with breast cancer | | |  |  |  |  |  |  |  |  |  |  |  |  |  |  |  |
| None | Ref. |  |  | Ref |  |  | Ref. |  |  | Ref. |  |  | Ref, |  |  | Ref. |  |
| 1^st^ degree relative | 0.585 | 0.391 - 0.876** |  | 0.560 | 0.373 - 0.842** |  | 0.844 | 0.557 - 1.278 |  | 0.905 | 0.594 - 1.379 |  | 0.858 | 0.535 - 1.377 |  | 0.926 | 0.574 - 1.496 |
| 2^nd^ degree relative | 0.688 | 0.516 - 0.916* |  | 0.665 | 0.497 - 0.889** |  | 0.992 | 0.730 - 1.348 |  | 1.006 | 0.738 - 1.371 |  | 1.007 | 0.715 - 1.418 |  | 1.018 | 0.719 - 1.441 |
| Other | 2.306 | 0.804 - 6.615 |  | 2.218 | 0.764 - 6.442 |  | 1.447 | 0.477 - 4.387 |  | 1.316 | 0.426 - 4.068 |  | 0.803 | 0.236 - 2.735 |  | 0.764 | 0.219 - 2.664 |
| I don’t know | 0.771 | 0.446 - 1.331 |  | 0.751 | 0.433 - 1.302 |  | 1.272 | 0.691 - 2.342 |  | 1.243 | 0.668 - 2.313 |  | 1.033 | 0.530 - 2.015 |  | 1.071 | 0.542 - 2.116 |
| *N* | 1052 |  |  | 1052 |  |  | 1052 |  |  | 1052 |  |  | 1052 |  |  | 1052 |  |

* *p*<0.05; ** *p*<0.01
