# Supplementary material for: The impact of temporal framing of breast cancer risk on perceptions of and motivations to engage with information about early diagnosis: Evidence from an online experiment
Source: PLoS One. 2025 Mar 26;20(3):e0320245. doi: 10.1371/journal.pone.0320245 (PMC11940651; doi:10.1371/journal.pone.0320245)
Supplement: Table S4 — (DOCX) [file pone.0320245.s004.docx]

Table S4. Logistic regressions on intentions and active interest (N=1052)

|  | **Model 1: Ordinal logistic regression on intentions to do self-checks** | | | | |  |  | **Model 2: Binary logistic regression on active interest reading more** | | | | |
| --- | --- | --- | --- | --- | --- | --- | --- | --- | --- | --- | --- | --- |
|  | Unadjusted model | |  | Adjusted model | |  | (%) | Unadjusted model | |  | Adjusted model | |
|  | OR | 95% CI |  | aOR | 95% CI |  |  | OR | 95% CI |  | aOR | 95% CI |
| Overall |  |  |  |  |  |  | (60.5) |  |  |  |  |  |
| Condition |  |  |  |  |  |  |  |  |  |  |  |  |
| Near future | Ref. |  |  | Ref |  |  | (59.9) | Ref. |  |  | Ref. |  |
| Distant future | 0.890 | 0.685 - 1.157 |  | 0.874 | 0.671 - 1.140 |  | (61.2) | 1.057 | 0.826 - 1.354 |  | 1.072 | 0.834 - 1.379 |
| Age |  |  |  |  |  |  |  |  |  |  |  |  |
| 40-45 years old | Ref. |  |  | Ref |  |  | (60.3) | Ref. |  |  | Ref. |  |
| 46-50 years old | 0.755 | 0.578 - 0.985* |  | 0.748 | 0.567 - 0.986* |  | (60.9) | 1.028 | 0.801 - 1.320 |  | 1.057 | 0.814 - 1.374 |
| Menopausal status |  |  |  |  |  |  |  |  |  |  |  |  |
| Premenopausal | Ref. |  |  | Ref |  |  | (60.6) | Ref. |  |  | Ref. |  |
| Postmenopausal | 0.754 | 0.469 - 1.212 |  | 0.873 | 0.534 - 1.426 |  | (60.4) | 0.995 | 0.641 - 1.544 |  | 0.937 | 0.590 - 1.488 |
| Numeracy question | | |  |  |  |  |  |  |  |  |  |  |
| Wrong | Ref. |  |  | Ref |  |  | (56.1) | Ref. |  |  | Ref. |  |
| Right | 1.231 | 0.866 - 1.752 |  | 1.160 | 0.810 - 1.661 |  | (61.5) | 1.248 | 0.902 - 1.726 |  | 1.307 | 0.936 - 1.827 |
| Education level |  |  |  |  |  |  |  |  |  |  |  |  |
| No A-levels | Ref. |  |  | Ref |  |  | (63.0) | Ref. |  |  | Ref. |  |
| A-levels or above | 0.883 | 0.592 - 1.318 |  | 0.814 | 0.539 - 1.229 |  | (60.2) | 0.890 | 0.612 - 1.292 |  | 0.826 | 0.560 - 1.219 |
| Paid employment |  |  |  |  |  |  |  |  |  |  |  |  |
| No | Ref. |  |  | Ref |  |  | (59.3) | Ref. |  |  | Ref. |  |
| Yes | 1.443 | 0.991 - 2.101 |  | 1.461 | 0.997 - 2.141 |  | (60.8) | 1.060 | 0.749 - 1.501 |  | 1.114 | 0.778 - 1.594 |
| Marital status |  |  |  |  |  |  |  |  |  |  |  |  |
| Single, divorced, separated or widowed | Ref. |  |  | Ref |  |  | (59.8) | Ref. |  |  | Ref. |  |
| Married or living with partner | 1.115 | 0.838 - 1.483 |  | 1.103 | 0.828 - 1.471 |  | (60.9) | 1.049 | 0.804 - 1.370 |  | 1.067 | 0.814 - 1.399 |
| Ethnicity |  |  |  |  |  |  |  |  |  |  |  |  |
| White British | Ref. |  |  | Ref |  |  | (58.8) | Ref. |  |  | Ref. |  |
| Other White background | 0.454 | 0.261 - 0.790** |  | 0.434 | 0.248 - 0.758** |  | (60.6) | 1.077 | 0.646 - 1.796 |  | 1.125 | 0.670 - 1.887 |
| Asian background | 0.361 | 0.169 - 0.770** |  | 0.387 | 0.180 - 0.831* |  | (86.8) | 4.619 | 1.786 - 11.943** |  | 4.690 | 1.806 - 12.181** |
| African/Black background | 0.326 | 0.138 - 0.770* |  | 0.336 | 0.141 - 0.799* |  | (67.9) | 1.477 | 0.661 - 3.302 |  | 1.576 | 0.697 - 3.563 |
| Mixed or other background | 1.179 | 0.529 - 2.628 |  | 1.248 | 0.557 - 2.795 |  | (71.0) | 1.711 | 0.779 - 3.758 |  | 1.836 | 0.830 - 4.061 |
| History with breast cancer | | |  |  |  |  |  |  |  |  |  |  |
| None | Ref. |  |  | Ref |  |  | (59.5) | Ref. |  |  | Ref. |  |
| 1^st^ degree relative | 1.185 | 0.732 - 1.919 |  | 1.147 | 0.704 - 1.870 |  | (60.2) | 1.030 | 0.648 - 1.637 |  | 1.064 | 0.666 - 1.699 |
| 2^nd^ degree relative | 1.402 | 0.992 - 1.981 |  | 1.367 | 0.963 - 1.941 |  | (63.4) | 1.177 | 0.842 - 1.645 |  | 1.205 | 0.858 - 1.691 |
| Other | 0.672 | 0.187 - 2.412 |  | 0.858 | 0.234 - 3.147 |  | (81.8) | 3.058 | 0.656 - 14.255 |  | 2.611 | 0.544 - 12.534 |
| I don’t know | 1.001 | 0.504 - 1.989 |  | 1.115 | 0.560 - 2.222 |  | (61.0) | 1.062 | 0.557 - 2.023 |  | 1.010 | 0.524 - 1.944 |
| *N* | 1052 |  |  | 1052 |  |  |  | 1052 |  |  | 1052 |  |

* *p*<0.05; ** *p*<0.05
