# Supplementary material for: The impact of temporal framing of breast cancer risk on perceptions of and motivations to engage with information about early diagnosis: Evidence from an online experiment
Source: PLoS One. 2025 Mar 26;20(3):e0320245. doi: 10.1371/journal.pone.0320245 (PMC11940651; doi:10.1371/journal.pone.0320245)
Supplement: Text S1 — (DOCX) [file pone.0320245.s005.docx]

### Text S1. Preliminary questionnaire

**Page 1: Welcome**

We would like to invite you to take part in this study on breast cancer, which is conducted by XXX. You should only take part if you want to. Before you decide whether to take part, it is important for you to read the following information carefully.

**What is the purpose of the study?**

This study investigates how women perceive the risk of breast cancer and how motivated they are to engage with preventative information.

**Why have I been invited?**

We are inviting women aged 45 to 50 years living in England to part in this survey.

**Do I have to take part?**

It is up to you to decide to join the study. Note that by completing each question, you are giving your consent for the information you provide to be used for research purposes. While all questions in the survey are mandatory and cannot be skipped, no personal information will be collected, meaning that your response will be anonymous and it will not be possible for you to be identified as an individual. You do not have to take part in the survey and you have the right to withdraw at any point during the survey without giving a reason. If you decide to withdraw, then the data you have provided up that point, will not be used for this project.

**What will I have to do if I take part?**

If you choose to take part in this study we would ask you to complete this online survey which should take around 3 minutes to complete. The survey is hosted by Qualtrics on a European server. We will download the data after the completion of the data collection and delete it from the server.

**What are the possible disadvantages and risks of taking part?**

The risk of taking part in this study is very low, as you will not be asked to provide sensitive personal data or involve in any dangerous activities. Furthermore, there are no physical or psychological risks for the participants, as there are no right or wrong answers to the questions in this voluntary and anonymous online survey.

**What are the possible benefits of taking part?**

Your participation in this study will help us to better understand attitudes towards breast cancer. you will receive an incentive from Prolific for completing the survey. Prolific will identify study participants, who completed the survey through individual URL redirects. Note that Prolific will not have access to the survey and your answers.

**Will my taking part in this project be kept confidential?**

Yes. All information which is collected about you during the course of the research is anonymous and not linked to any of your personal details. This means that you cannot be recognised from the information you provide.

**What will happen to the results of the research project?**

We hope to report the findings from the study in an academic journal. You will not be identified in any reports or publications from the study. Additionally, the anonymized data from this study will be made publicly available on the website Open Science Framework (https://www.osf.io), which allows researchers to share and collaborate research, after the publication in the academic journal.

**What do I need to do?**

Once you’ve read this information and if you’re happy to do so, please continue to answer the survey questions.

**If you have any questions please contact XXX**

Thank you for reading this information and for considering to take part in this research study.

**Page 2: Consent form**

Thank you for considering taking part in this research. If you have any questions arising from the Information explanation already given to you, please ask the researcher before you decide whether to join in.

I confirm that I understand that by ticking/initialling each box below I am consenting to this element of the study. I understand that it will be assumed that unticked/uninitialed boxes means that I DO NOT consent to that part of the study. I understand that by not giving consent for any one element that I may be deemed ineligible for the study.

|  | Tick Box |
| --- | --- |
| I confirm that I have read and understood the information sheet for the study above. I have had an opportunity to consider the information and what will be expected of me. I have also had the opportunity to ask questions. |  |
| I understand that my participation is voluntary and that I am free to withdraw at any time without giving a reason |  |
| I understand that my data gathered in this study will be stored anonymously and securely.  It will not be possible to identify me in any publications. |  |
| I understand and agree with the publication of the anonymized data. I am also informed that the anonymized data will be available on open access on Open Science Framework. |  |
| I understand that the data will not be made available to any commercial organisations but is solely the responsibility of the researchers undertaking this study. |  |
| I voluntarily agree to take part in this study. |  |

**Page 3: Demographic Information (filter questions)**

1. What is your sex?
   - Male (exclude from survey)
   - Female
   - Transgender (exclude from survey)
   - Non-binary (exclude from survey)
   - Other (exclude from survey)
   - I don’t want to say (exclude from survey)
2. What is your age?
   - Younger than 40 years old (exclude from survey)
   - 40-45 years
   - 45-50 years
   - Older than 50 years old (exclude from survey)
3. What is your menopausal status? (A woman is said to be in menopause (= postmenopausal) when she hasn't had a period for 12 months in a row.)
   - Premenopausal
   - Postmenopausal
   - I don’t know (exclude from survey)
4. Which of the following numbers represents the biggest risk of getting a disease?
   - 1/10
   - 1/100
   - 1/1000
   - I don’t know

**Page 4: Perception of the risk messages**

[For women aged 40-50] Please read the following risk messages about developing breast cancer.

- - There is a 2-3% probability of developing breast cancer in the next 20 years.
  - 43% of women diagnosed with breast cancer were older than 65 years.
  - 1 in 29 women in the age between age of 60 and 70 is diagnosed with breast cancer

1. Given that all three messages refer to the same risk of women being diagnosed with breast cancer, which one is easier to understand?
   - There is a 2-3% probability of developing breast cancer in the next 20 years.
   - 43% of women diagnosed with breast cancer were older than 65 years.
   - 1 in 29 women in the age between age of 60 and 70 is diagnosed with breast cancer
   - All three are easy to understand
   - None of them is easy to understand
2. Given that all three messages refer to the same risk of women being diagnosed with breast cancer, which one is easier to imagine?
   - There is a 2-3% probability of developing breast cancer in the next 20 years.
   - 43% of women diagnosed with breast cancer were older than 65 years.
   - 1 in 29 women in the age between age of 60 and 70 is diagnosed with breast cancer
   - All three are easy to imagine
   - None of them is easy to image
3. Given that all three messages refer to the same risk of women being diagnosed with breast cancer, which one is more motivational to engage in behaviours to prevent breast cancer?
   - There is a 2-3% probability of developing breast cancer in the next 20 years.
   - 43% of women diagnosed with breast cancer were older than 65 years.
   - 1 in 29 women in the age between age of 60 and 70 is diagnosed with breast cancer
   - All three are motivational
   - None of them is motivational
4. Given that all three messages refer to the same risk of women being diagnosed with breast cancer, which one should be used by the NHS for communication?
   - There is a 2-3% probability of developing breast cancer in the next 20 years.
   - 43% of women diagnosed with breast cancer were older than 65 years.
   - 1 in 29 women in the age between age of 60 and 70 is diagnosed with breast cancer
   - All three should be used
   - None of them should be used

**Page 6: About yourself**

1. Have you obtained A-levels or above
   - Yes
   - No
2. Are you currently in paid employment
   - Yes
   - No
3. What is your marital status
   - Single
   - Married or living with partner
   - Divorced, separated or widowed
4. Which of the following describes your ethnic group best?
   - White British
   - Other White background
   - African/Black background
   - Asian background
   - Hispanic background
   - Mixed background
   - Other

**Page 7: Thank you for participating in this survey!**

If you are interested in breast cancer related resources:

- Breast cancer symptoms and signs: https://breastcancernow.org/information-support/have-i-got-breast-cancer/signs-symptoms-breast-cancer
- Support for individuals living with breast cancer: https://breastcancernow.org/information-support/facing-breast-cancer
- Forum: https://forum.breastcancernow.org/

Breast cancer prevention: https://preventbreastcancer.org.uk
