# Supplementary material for: The impact of temporal framing of breast cancer risk on perceptions of and motivations to engage with information about early diagnosis: Evidence from an online experiment
Source: PLoS One. 2025 Mar 26;20(3):e0320245. doi: 10.1371/journal.pone.0320245 (PMC11940651; doi:10.1371/journal.pone.0320245)
Supplement: Text S2 — (DOCX) [file pone.0320245.s006.docx]

### Text S2. Main questionnaire

**Page 1: Welcome**

We would like to invite you to take part in this study on breast cancer, which is conducted by XXX. You should only take part if you want to. Before you decide whether to take part, it is important for you to read the following information carefully.

**What is the purpose of the study?**

This study investigates how women perceive the risk of breast cancer and how motivated they are to engage with preventative information.

**Why have I been invited?**

We are inviting women aged 45 to 50 years living in England to part in this survey.

**Do I have to take part?**

It is up to you to decide to join the study. Note that by completing each question, you are giving your consent for the information you provide to be used for research purposes. While all questions in the survey are mandatory and cannot be skipped, no personal information will be collected, meaning that your response will be anonymous and it will not be possible for you to be identified as an individual. You do not have to take part in the survey and you have the right to withdraw at any point during the survey without giving a reason. If you decide to withdraw, then the data you have provided up that point, will not be used for this project.

**What will I have to do if I take part?**

If you choose to take part in this study we would ask you to complete this online survey which should take around 4 minutes to complete. The survey is hosted by Qualtrics on a European server. We will download the data after the completion of the data collection and delete it from the server.

**What are the possible disadvantages and risks of taking part?**

The risk of taking part in this study is very low, as you will not be asked to provide sensitive personal data or involve in any dangerous activities. Furthermore, there are no physical or psychological risks for the participants, as there are no right or wrong answers to the questions in this voluntary and anonymous online survey.

**What are the possible benefits of taking part?**

Your participation in this study will help us to better understand attitudes towards breast cancer. you will receive an incentive from Prolific for completing the survey. Prolific will identify study participants, who completed the survey through individual URL redirects. Note that Prolific will not have access to the survey and your answers.

**Will my taking part in this project be kept confidential?**

Yes. All information which is collected about you during the course of the research is anonymous and not linked to any of your personal details. This means that you cannot be recognised from the information you provide.

**What will happen to the results of the research project?**

We hope to report the findings from the study in an academic journal. You will not be identified in any reports or publications from the study. Additionally, the anonymized data from this study will be made publicly available on the website Open Science Framework (https://www.osf.io), which allows researchers to share and collaborate research, after the publication in the academic journal.

**What do I need to do?**

Once you’ve read this information and if you’re happy to do so, please continue to answer the survey questions.

**If you have any questions please contact XXX**

Thank you for reading this information and for considering to take part in this research study.

**Page 2: Consent form**

Thank you for considering taking part in this research. If you have any questions arising from the Information explanation already given to you, please ask the researcher before you decide whether to join in.

I confirm that I understand that by ticking/initialling each box below I am consenting to this element of the study. I understand that it will be assumed that unticked/uninitialed boxes means that I DO NOT consent to that part of the study. I understand that by not giving consent for any one element that I may be deemed ineligible for the study.

|  | Tick Box |
| --- | --- |
| I confirm that I have read and understood the information sheet for the study above. I have had an opportunity to consider the information and what will be expected of me. I have also had the opportunity to ask questions. |  |
| I understand that my participation is voluntary and that I am free to withdraw at any time without giving a reason |  |
| I understand that my data gathered in this study will be stored anonymously and securely.  It will not be possible to identify me in any publications. |  |
| I understand and agree with the publication of the anonymized data. I am also informed that the anonymized data will be available on open access on Open Science Framework. |  |
| I understand that the data will not be made available to any commercial organisations but is solely the responsibility of the researchers undertaking this study. |  |
| I voluntarily agree to take part in this study. |  |

**Page 3: Demographic Information (filter questions)**

1. What is your sex?
   - Male (exclude from survey)
   - Female
   - Transgender (exclude from survey)
   - Non-binary (exclude from survey)
   - Other (exclude from survey)
   - I don’t want to say (exclude from survey)
2. What is your age?
   - Younger than 40 years old (exclude from survey)
   - 40-45 years
   - 45-50 years
   - Older than 50 years old (exclude from survey)
3. What is your menopausal status? (A woman is said to be in menopause (= postmenopausal) when she hasn't had a period for 12 months in a row.)
   - Premenopausal
   - Postmenopausal
   - I don’t know (exclude from survey)
4. Which of the following numbers represents the biggest risk of getting a disease?
   - 1/10
   - 1/100
   - 1/1000
   - I don’t know

**Page 4: Breast Cancer Risk Factors – perception**

1. Please indicate your level of knowledge about breast cancer risk factors:
   - Very knowledgeable
   - Somewhat knowledgeable
   - Not knowledgeable
2. Which of the following do you think are risk factors for breast cancer? (Select all that apply)
   - Family history of breast cancer
   - Age
   - Hormone replacement therapy
   - Excess body weight and obesity
   - Alcohol consumption
   - Lack of physical activity
3. How often do you actively seek information about breast cancer prevention?
   - Never
   - Rarely (once every 5 years)
   - Occasionally (once every year)
   - Frequently (once every 6 months)
   - Very often (every month)
4. How likely are you to share breast cancer prevention information with others? (for example on social media, through email, or in a conversation)
   - Not likely at all
   - Unlikely
   - Neutral
   - Likely
   - Very likely
5. How motivated are you to prevent breast cancer?
   - Not motivated at all
   - Slightly motivated
   - Moderately motivated
   - Highly motivated
   - Extremely motivated

**Page 5: About breast cancer – experimental manipulation**

Thank you for filling out the initial questions. We would now like to introduce the main part of the survey.

Breast cancer …. (depends on the results from the preliminary study)

- Near-future condition: There is a 1.4% risk of developing breast cancer in the next 10 years
- Distant-future condition: There is a 2-3% risk of developing breast cancer in the next 20 years

1. A breast self-exam can help you detect breast cancer earlier when it is easier to treat.

"In the context of this information, how important is it for you to learn how to conduct a breast self-exam?"

- - Not important at all
  - Slightly important
  - Moderately important
  - Highly important
  - Extremely important

1. Would you be willing to engage in preventive behaviours to mitigate developing breast cancer?
   - Definitely not
   - Probably not
   - Yes, probably
   - Yes, definitely
2. How temporally imminent or distant do you think the likelihood is of breast cancer occurring to you? (1 =very imminent vs. 7 =very distant)
3. How serious do you think the health consequences of breast cancer are? (1 =not serious at all vs. 7 =very serious), respectively.

**Page 6: Behavioural proxy question**

1. Before continuing with the survey, would you be interested in reading more about self-examination?

- Yes, I would like to read about self-examination before continuing with the survey
- No, I want to skip the list and finish the survey

**Page 7: Additional information about self-examination**

The NHS Breast Screening Programme has produced a 5-point plan for being breast aware:

- know what's normal for you
- look at your breasts and feel them
- know what changes to look for
- report any changes to a GP without delay
- attend routine screening if you're aged 50 to 70

Look at your breasts and feel each breast and armpit, and up to your collarbone. You may find it easiest to do this in the shower or bath, by running a soapy hand over each breast and up under each armpit.

You can also look at your breasts in the mirror. Look with your arms by your side and also with them raised.

See a GP if you notice any of the following changes:

- a change in the size, outline or shape of your breast
- a change in the look or feel of the skin on your breast, such as puckering or dimpling, a rash or redness
- a new lump, swelling, thickening or bumpy area in one breast or armpit that was not there before
- a discharge of fluid from either of your nipples
- any change in nipple position, such as your nipple being pulled in or pointing differently
- a rash (like eczema), crusting, scaly or itchy skin or redness on or around your nipple
- any discomfort or pain in one breast, particularly if it's a new pain and does not go away (although pain is only a symptom of breast cancer in rare cases)

**Page 8: Questions about self-examination article**

1. What should you do if you notice a new lump, swelling, or bumpy area in one breast or armpit that was not there before?
   - Report the changes to your GP without delay.
   - Ignore the changes, as they are likely not significant.
2. What is the recommended action if you notice a change in the look or feel of the skin on your breast, such as puckering or dimpling, a rash or redness?

- Monitor the changes for a few weeks to see if they resolve on their own.
- See a GP without delay to have the changes assessed.

1. What age group is recommended to attend routine breast cancer screening according to the NHS Breast Screening Programme?

- Women aged 40 to 70 years old.
- Women aged 50 to 70 years old.

**Page 9: Further questions about knowledge**

1. How often do you think about the long-term consequences of your health-related behaviours?
   - Never
   - Rarely
   - Occasionally
   - Frequently
   - Often
2. How often do you think about the immediate consequences of your health-related behaviours?
   - Never
   - Rarely
   - Occasionally
   - Frequently
   - Always

**Page 10: About yourself**

13. Have you obtained A-levels or above

- Yes
- No

1. Are you currently in paid employment

- Yes
- No

1. What is your marital status

- Single
- Married or living with partner
- Divorced, separated or widowed

1. Which of the following describes your ethnic group best?

- White British
- Other White background
- African/Black background
- Asian background
- Hispanic background
- Mixed background
- Other

1. Do you have family history of breast cancer?
   - Yes
   - No
   - I don’t know
2. If yes, what was their relationship to you?
   - 1^st^ degree relative (parents, siblings, children)
   - 2^nd^ degree relative (grandparents, grandchildren, uncle, aunt, nephew, niece, half-sibling)
   - Other

**Page 11: Thank you for participating in this survey!**

If you are interested in breast cancer related resources:

- Breast cancer symptoms and signs: https://breastcancernow.org/information-support/have-i-got-breast-cancer/signs-symptoms-breast-cancer
- Support for individuals living with breast cancer: https://breastcancernow.org/information-support/facing-breast-cancer
- Forum: https://forum.breastcancernow.org/
- Breast cancer prevention: https://preventbreastcancer.org.uk/
